# Characterization of white chocolate enriched with co‐encapsulated *Lactobacillus acidophilus* (*La‐5*) and rose hip shell fruit extract: Characterization, probiotic viability during storage, and in vitro gastrointestinal digestion

**DOI:** 10.1002/fsn3.3805

**Published:** 2023-11-20

**Authors:** Zohreh Didar

**Affiliations:** ^1^ Department of Food Science and Technology, Neyshabur Branch Islamic Azad University Neyshabur Iran

**Keywords:** Co‐encapsulation, *Lactobacillus acidophilus*, rose hip fruit shell extract

## Abstract

This research focused on the production of a new kind of probiotic chocolate containing co‐encapsulated *Lactobacillus acidophilus* (*La‐5*) bacteria and rose hip shell fruit extract. Several properties of chocolate samples, including rheological, textural, thermal properties, particle size distribution, color indices, total phenolic and anthocyanin magnitude, antioxidant potential, and Raman spectroscopy were performed. The prepared white chocolates were assessed for the survival of the probiotic cell and the stability of anthocyanins and phenolic components in different storage times (until 90 days) and different storage temperatures (at 4 and 25°C). Observations imply that both temperature and duration of storage had an impact on the extent of survival of probiotics as well as stability of total phenolic content (TPC) and anthocyanin content (*p* < .05). During in vitro gastrointestinal circumstances, the extent of survival of *L. acidophilus*, in two chocolate matrixes, was assessed. At the end of gastric and intestinal condition, the log of viable cells was 7 and 6, respectively. The magnitude of the bioaccessibility of anthocyanin and phenolic components was 81% and 78%, respectively. Sensory evaluation affirmed that there was no remarkable variation between samples in terms of overall acceptance.

## Graphical abstract

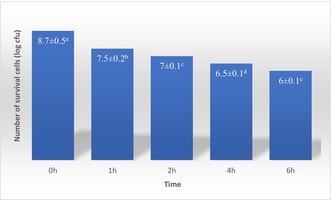

## 1 INTRODUCTION

Rosaceae Juss. is from the Angiospermae family. The genus *Rosa* L. is composed of approximately 200 species. The fruits of *Rosa* L. (Rosae pseudo‐fructus) have been applied as raw substances for preparing medicinal products for a long time. Fruits have been reported to compose several categories of biologically active components (Butkevičiūtė et al., 2022) so it has wide usage in medicine and the food technology (Koczka et al., 2018). Several biological activities of fruit are reported like anti‐inflammatory (Wenzig et al., 2008), antioxidant (Barros et al., 2011; Bozhuyuk et al., 2021), and antiproliferative (Tumbas et al., 2012) impacts. Clinical research has indicated that the fruit diminishes the signs of osteoarthritis (Cosmulescu et al., 2017), protects the kidneys against oxidative stress (Shahidi, 2004), and has various biological impacts like anti‐inflammatory, antimicrobial, anticancer, and antidiabetic (Mármol et al., 2017). Wild Rose canina fruit possesses several bioactive compounds (Igual et al., 2021). Various phenolic components were reported in the fruit of the genus *Rosa* L. include: caffeic acid, chlorogenic acid, quercetin, quercitrin, (+)‐catechin, (−)‐epicatechin, (−)‐epicatechin gallate, rutin, phloridzin, and kaempferol‐3‐*O*‐glycoside (Liaudanskas et al., 2021). Mármol et al. (2017) reported the presence of various hydrosoluble antioxidants (Gallic acid, vanillic acid, Ellagic acid, Quercetin, Catechin, Kaempferol, Myricetin, and Rutin) and lipid‐soluble antioxidants (B‐ carotene, Lycopene, Tocopherol) in rose hips (Mármol et al., 2017).

Several applications of the fruit of *Rosa* L. are reported such as in beverages, jellies, and jams. Some researches were focused on substituting *Rosa* L. fruit instead of some food additives (Jiménez et al., 2017). Lately, the fruit has been applied in the production of some food products such as probiotic beverages and yogurts, and in soups (Su et al., 2007). Other studies include the usage of *R. pimpinellifolia* fruit extract in yogurt and assessment the final quality of yogurt (Bobinait et al., 2012), marshmallow candy (Ghendov‐Moșanu, 2018), and ice cream (Zandilak & Yazdanpanah, 2018). Igual et al. (2021) used the encapsulated powder of wild *Rosa canina* fruit to produce corn extrudates and reported the positive impact on its functional value.

The existence of live probiotic microorganisms which is in sufficient concentrations (more than 10^7^ CFU mL^−1^) in food materials resulted in useful health impacts on consumers (WoldemariamYohannes et al., 2020). *Lactobacillus* is a major probiotic bacterium (Shakibaie et al., 2017). A remarkable parameter concerning probiotic foods is the extent of survival and the amount of the probiotic microorganisms prior to consumption and at the time of expiry. Encapsulation of probiotics is an alternative approach for extending viability and functionality during the storage of probiotic foods. Various studies focused on production of probiotic products with desirable survival of probiotic bacteria in the finished product and during the storage period. Mahmoodi Pour et al. (2022) assessed applying various emulsions containing probiotic bacteria (*Lactobacillus rhamnosus* and *Lactobacillus plantarum*) for the production of probiotic yogurts. Accordingly, multilayer emulsion containing encapsulated probiotic bacteria showed the best survival of probiotics (Mahmoodi Pour et al., 2022). Barat and Ozcan (2018) focused on the production of probiotic fermented milk beverages with various fruit matrices (black mulberry, red grape, and cornelian cherry) and concluded that produced probiotic beverage maintains a high magnitude of probiotic bacteria and their therapeutic activity during storage (Barat & Ozcan, 2018).

Co‐encapsulation implies the guarding of components from harsh circumstances. However, the release of encapsulated components in the gut is necessary to access their health profits. Co‐encapsulation of probiotic cells and plant extracts is focused on by some studies. Silva et al. (2022) evaluated the encapsulation of probiotics and guaraná extracts. Accordingly, guaraná peel extract (GPE) extends the development of probiotic cells (Silva et al., 2022). Mirmazloum et al. (2021) assessed co‐encapsulated *Ganoderma lingzhi* extract and probiotic bacteria. Accordingly, remarkable modification in the survival of probiotic cell under simulated gastrointestinal (SGI) conditions has been reported (Mirmazloum et al., 2021).

Chocolate is becoming a common carrier for delivering probiotic cells to the gut (Homayouni Rad et al., 2016). Dark, milk, and white chocolate are the main types of chocolate (Possemiers et al., 2010). Due to the high popularity of chocolate and its health beneficial impacts, the enrichment of various kinds of chocolate with probiotics has a high market appeal (Min et al., 2019). Using chocolate for carrying probiotics has not been extensively assessed (Min et al., 2019). The carrier has a remarkable effect on the effectiveness of probiotics (Hossain et al., 2021).

This research focused on co‐encapsulation of *Lactobacillus acidophilus* and rose hip fruit shell extract and its application in the white chocolate formulation and characterization of white chocolate properties.

## 2 MATERIALS AND METHODS

In the spring of 2022, samples of the flower of *Rosa canina* L. from Neyshabur region were collected and authenticated by the Department of Botany of the Islamic Azad University, Neyshabur Branch, Iran. After identification, the fruit samples were collected from this area in autumn and immediately transferred to the freezer at −20°C until further analysis (Saidi et al., 2014). All chemicals used in this study were of analytical grade.

### 2.1 Preparation of *Rosa* L. fruit

The *Rosa* L. fruits were grounded after removing the seeds and internal lints (Zandilak & Yazdanpanah, 2018) and the shells obtained. Thereafter, for preparation of *Rosa* L. fruit extract, 0.5 g of dried *Rosa* L. shell was mixed with ethanol (40% (v/v, 10 mL)) in an ultrasonic bath (Euronda, Model Eurosonic 4D, Italy) (50 min, 25°C). The obtained extract was filtered (Shahbazi et al., 2022). Ethanol 40% (v/v) was used for washing the mass on the filter. Then, the volume of filtered extract reached to 10 mL by adding ethanol 40% (v/v) (Butkevičiūtė et al., 2022). Thereafter, the evaporation was performed by a rotary evaporator (40 ± 1°C) (Justine et al., 2019).

### 2.2 Preparation of *L. acidophilus*

For activation of probiotic bacteria, the lyophilized *L. acidophilus* was inoculated into MRS broth (De Man, Rogosa, and Sharpe) and incubation was carried out at 37°C, 24 h. Thereafter, centrifugation was performed (3000 *g*, 4°C, 10 min), and harvested cells were washed two times with distilled water. Thereafter, the cells (about 10^10^ CFU mL^−1^) were blended with sterile NaCl solution (0.9% w/v) and applied for encapsulation (Bakhtiyari et al., 2022).

### 2.3 Simultaneous encapsulation of probiotics and rose hip extract

For fabrication of the coacervates containing probiotics and rose hip extract, about 1.5 g of the probiotic was mixed in 1.5 g of rose hip extract (1449 *g*, 1 min). Thereafter, gelatin solution (150 mL, 2.5% w/w) was added and mixed (4025 *g*, 60 s). Gum Arabic solution (150 mL, 2.5% w/w) was mixed into the solution and stirred, thereafter, the pH was regulated to 4.2 by citric acid (5 M). Then, distilled water (600 mL) was mixed and stirred until the temperature reached 10°C (Silva et al., 2022). The mixture was frozen (−20°C, overnight) and then freeze‐dried (Model DW1.0–110; Heto‐Holten A/S) at−55°C (Hossain et al., 2022).

### 2.4 Morphological assessment of coacervates

The coacervates was assessed for morphology properties by applying an SEM scanning electron microscopy (SEM; proX phenom).

### 2.5 Assess the total phenolic content (TPC)

TPC was evaluated following the approach of Silva et al. (2022). First, the sample (0.25 mL) was mixed with distilled water (2 mL) and Folin–Ciocalteu reagent (0.25 mL). Thereafter, the saturated sodium carbonate (0.25 mL) was added to the solution, and vortexed and for completing the reaction, incubation was performed (in a water bath at 37°C, 30 min). The absorbance was read at 750 nm UV–Vis spectrophotometer (Jenway, 6300, UK). Gallic acid was used as reference for the determination of TPC.

### 2.6 Encapsulation efficiency (EE) of phenolic content

Encapsulation efficiency was assessed according the approach described by de Souza et al. (2018). coacervate (0.1 g) was mixed with distilled water (5 mL) and vortexed for 1 min. Thereafter, centrifugation was performed (6603 *g*, 5 min). The magnitude of TPC was calculated in the supernatant section as explained in previously. The EE for phenolics from rose hip extract was calculated by applying the following equation:

(1) $$\mathrm{EE}\;\left(\%\right)=\frac{\text{Bioactive coacervate}‐\text{Bioactive surface}}{\text{Bioactive initial}}\times 100$$

where, bioactive coacervates: total amount of phenolics in coacervates; bioactive surface: total magnitude of phenolics on the surface of coacervates; bioactive initial: total amount of phenolics added to the polymers for encapsulation (de Souza et al., 2018).

### 2.7 Anthocyanin content

The measurement of anthocyanin content was carried out based on the approach of Brito et al. (2014). The investigation of total anthocyanin content (TAC) was accomplished by the pH differential approach at 510 and 700 nm in buffers at pH 1.0 and 4.5. Anthocyanin amount is reported as mg cyanidin 3‐glucoside equivalents/g dry mass and assessed by the following formula:

(2) $$\mathrm{TAC}\;\left(\mathrm{mg}\;g^{-1}\right)=\frac{A\times \mathrm{MW}\times \mathrm{DF}\times 10^{5}}{\varepsilon \times 1}$$

where, *A* = (*A*_510 nm_ − *A*_700 nm_) pH 1.0 − (*A*_510 nm_ − *A*_700 nm_) pH 4.5; MW (molecular weight) = 449.2 g mol^−1^; DF = dilution factor; 1 = cuvette pathlength in cm; *ε* = 26,900 L mol.cm^−1^, molar extinction factor for cyanidin 3‐O‐β‐d‐glucoside. Jenway‐ 6300‐ UV–Vis (UK) spectrophotometer was used (Brito et al., 2014).

### 2.8 Encapsulation efficiency of total anthocyanin content

The total anthocyanin magnitude of the microcapsules was assessed by applying an approach described by Seke et al. (2022). First destabilization of the microcapsule was done by homogenizing using sodium citrate (5%, 10 mL) to reach complete dissolution. The total anthocyanin content (TAC) was determined via a pH differential approach outlined previously. The encapsulation efficiency was assessed by Equation (1) (De Cássia Sousa Mendes et al., 2021).

As follows,

(3) $$\mathrm{EE}\;\left(\%\right)=\frac{\text{Anthocyanin content in beads}}{\text{Anthocyanin content in extract}}\times 100$$

### 2.9 Enumeration of probiotic

The specific amount (100 μL) was withdrawn and diluted serially. Then, incubation in MRS (De Man, Rogosa, and Sharpe) agar was performed. After the incubation (37°C in an anaerobic jar, 48 h), an enumeration of viable probiotics was performed. Results were depicted as the number of colony‐forming units (CFU) per milligram or gram (Silva et al., 2022).

### 2.10 Encapsulation efficiency of probiotic

The final efficiency of the microcapsules was calculated as the approach outlined by Afzaal et al. (2022).

$$\text{Encapsulation yield}=\frac{N}{N_{0}}\times 100$$

### 2.11 Antioxidant activity

The DPPH approach was accomplished as outlined by Helal et al. (2022). First, a specific amount of sample (200 μL) was blended with 0.1 mmol L^−1^ methanolic solution of DPPH (2 mL). After incubation (30 min at dark condition), the absorbance was read at 517 nm. For calibration curve, ascorbic acid was applied and the results are depicted as mg ascorbic acid equivalent/100 g of samples (Helal et al., 2022).

### 2.12 Preparation of probiotic white chocolate samples

For preparing white chocolate the following materials at specific amounts were used. For production of 1 kg of chocolate, the amount of ingredients was as follows: sugar (497.6 g), cocoa butter (348.4 g), whole milk powder (149.3 g), sunflower lecithin (4.5 g), vanilla powder (0.2 g; Lončarević et al., 2018). First, 20% of the total cocoa butter was melted and blended with sugar and whole milk powder until formation, a homogeneous mixture meantime was heated to 40°C. Thereafter, a pre‐refining step of the chocolate mass was performed by a pilot‐scale 3‐roll refiner (Lehmann) and thereafter blended and warmed to 50°C. The next stage was dry conching (45 min), and the residual of cocoa butter and soy lecithin were mixed. The total conching step was done at 60°C and 360 min. The inclusion of encapsulated probiotic powder 1% (w/w) was carried out after the conching step (Hossain et al., 2022). Seke et al. (2022) at 32–33°C and mixing (5 min). Then, tempering was accomplished at three stages including 33–35, 24–25, and 25–26°C, respectively. Molding and vibration steps were done at 27–30°C. Cooling was done at 5°C for 20 min. The produced samples were stored (13–15°C in cold and dark circumstances) before analysis (Toker et al., 2018).

### 2.13 Extraction procedure of chocolate samples for antioxidant and TPC analysis

For preparing chocolate extracts, 0.5 g of chocolate was ground and defatted with n‐hexane (50 mL) at room temperature (30 min). Thereafter, the residual solvent was evaporated from defatted solids by air‐drying (24 h). For extraction of antioxidants, 2.0 g of defatted chocolate samples were blended with 10 mL of acetone–water–acetic acid with a ratio equal to 70:29.8:0.2, v/v/v, and was shaken for 30 min. Extractions were performed twice at room temperature. Finally, extracts were filtered through a polytetrafluorethylene syringe filter and stored in a refrigerator until the antioxidant activity and total phenolic content analysis (Poliński et al., 2021).

## 3 PHYSICOCHEMICAL ANALYSIS OF CHOCOLATE SAMPLES

### 3.1 Measurement of color indices

The color of white chocolate samples was determined three times using MINOLTA Chroma Meter CR‐400 (Minolta Co., Ltd.; Lončarević et al., 2018).

### 3.2 Melting properties

The melting behavior of the white chocolate samples was assessed using DSC (Differential Scanning Calorimeter; TA Q20, TA Instruments) following the approach of Glicerina et al. (2013). The amount of sample was 5 mg and heating profile was 0–60°C at 10°C min^−1^. The determined parameters were onset temperature (*T*_onset_), peak temperature (*T*_peak_), and energy required for the complete melting of the samples (Δ*H*; Glicerina et al., 2013).

### 3.3 Rheological properties

A rheometer (MCR 302; Anton Paar) was applied for the assessment of the flow behavior of the melted chocolate samples at 40°C. Casson viscosity (Pas) and Casson yield stress (Pa) were determined as follows:

$$\tau^{0.5}=\tau_{0}^{0.5}+\eta_{\mathrm{pl}.\gamma \cdot^{n}}$$

where, *τ* is shear stress (Pa), ·_γ_ is the shear rate (*s* ^−1^), *τ*_0_ is the yield stress (Pa), and *η*_pl_ is plastic viscosity (Pas).

### 3.4 Microstructural examination

The structure of the chocolate samples was analyzed by SEM technique, applying a phenom proX SEM (Netherlands), 500–1000 magnification.

### 3.5 Particle size and zeta potential

The particle size distribution, polydispersity index (PDI), and zeta potential were determined by A Zetasizer Nano ZS90 (Malvern Instruments Ltd.; Didar & Hesarinejad, 2022).

### 3.6 Texture analysis

For investigation of the texture of chocolate samples, a TA.XT plus texture analyzer (Stable Micro Systems) was applied. The analysis was set as the single penetration event and at the temperature of 22 ± 1°C. The hardness of the samples was measured by penetrating an aluminum probe into the chocolate sample. The analysis condition was as follows: probe diameter, 2 mm; penetration rate, 2 mm s^−1^; and penetration depth, 5 mm (Lapčíková et al., 2022).

### 3.7 Raman spectroscopy

The measurements were performed by applying a Unicorn (South Korea) RAMAN spectrometer. The tests were performed with a laser of wavelength *λ*_0_ = 785 nm, of maximum power, *P*_max_ = 25 mW, an acquisition time of 100 s, and an addition of two spectra (50–3470 cm^−1^; el Hadri et al., 2022).

### 3.8 Thermogravimetric analysis (TGA)

The analyses were performed by applying a thermogravimetric analyzer (TA, model Q600; Ostrowska‐Ligęza et al., 2018).

### 3.9 Storage study

All chocolate samples were packed in aluminum foil (0.2 mm thickness) and stored at two different temperatures (4 and 25°C). The probiotic viability, TPC, and TAC stability were assessed (Islam et al., 2022).

### 3.10 Probiotic viability in white chocolate during storage

The viability of probiotic bacteria in white chocolate samples maintained at different temperatures (4 and 25°C) for 90 days was determined at days 0, 7, 30, 60, and 90. For this reason, melting of samples was performed (37°C, 10 min) prior to determination of the total viable count. The total viable count (Log_10_ CFU mL^−1^) was determined by the common plate count method. The 1 mL of samples was poured into sterile saline solution (0.9% NaCl w/v) (in sterile condition) and serial dilution was done (up to five folds). Thereafter, 10–15 mL of agar media (63.5 g L^−1^ distilled water) was plated. The solidified plates were incubated (37°C, 48 h). The final step was enumeration and reporting the number of colonies as colony‐forming units (CFU g^−1^; Islam et al., 2022).

### 3.11 Probiotic viability, TPC, and TAC content during in vitro gastrointestinal digestion

The cell survival rate of the probiotic strain (*Lactobacillus acidophilus La‐5*) on gastrointestinal digestion of chocolate samples was evaluated based on the approach of Islam et al. (2022) and Minekus et al. (2014). For this reason, five sections of chocolate were blended with five sections of simulated gastric fluid (SGF) to obtain a finished sample and SGF ratio of 50:50 (v/v), followed by the addition of 1 section double distilled water. Porcine pepsin was mixed into the solution to reach 2000 U mL^−1^, followed by the blending of CaCl_2_ to gain 0.075 mM in the total digestion solution. Also, 1 M HCl was applied for lowering the pH to 3.0. Lastly, the fabricated blend was incubated (2 h, 37°C). Cell viability in subjecting to intestinal juice was determined by applying the approach of Islam et al. (2022). Trypsin (0.1 g) and bile salts (1.8 g) were blended into a sterile solution of sodium bicarbonate (1.1 g) and of sodium chloride (0.2 g) in 100 mL of distilled water. Sodium hydroxide (0.5 M) was applied for regulating the pH to 8.0. For sterilization of simulated gastric and intestinal juices, filtration by a 0.45‐μm membrane (Pall) was performed. After fermentation, centrifugation (500 *g*, 5 min) and washing three times in phosphate‐buffered saline (PBS, pH 7.0) were done. Then, the sample was examined against the fabricated medium and incubated in a shaking incubator for 4 h at 37°C. At the end, cell survival rate was evaluated on MRS agar medium three times, and a specific amount (1 mL) from each incubated fluid (0, 1, 2, 4, and 6 h) was gathered to be diluted with 9 mL of 0.2 M sterile phosphate buffer (pH 7; Himedia). The results were expressed as log CFU mL^−1^.

For assessment the release behavior of TPC and TAC in vitro condition, the methods outlined in section 0.5 and 0.7 (material and methods) were performed. For each digested chocolate, a specific amount was gathered at the finish step of the digestion and evaluated for TPC (Silva et al., 2022) and TAC content (Kanha et al., 2021).

The magnitude of recovery after the gastric phase and bioaccessibility after the intestinal phase was assessed by applying Equations 2 and 3, respectively. Furthermore, after a particular time, a specific amount (2 mL) of the reaction solution was gathered (10, 30, 60, 90, and 120 min) and the magnitude of release on simulated gastric and intestinal circumstances was determined (Seke et al., 2022).

(4) $$\text{Recovery}\;\left(\%\right)=\frac{\text{Anthocyanin content in gastric digesta microcapsule}}{\text{Anthocyanin content in undigested microcapsule}}\times 100$$

(5) $$\mathrm{Bio}\;\text{accessibility}\;\left(\%\right)=\frac{\text{Anthocyanin content in intestinal digesta microcapsule}}{\text{Anthocyanin content in undigested microcapsule}}\times 100$$

### 3.12 Scanning electron microscopy

The morphology of both control and enriched chocolates were analyzed with scanning electron microscopy (SEM; proX phenom).

### 3.13 Sensory characterization of chocolate samples

Sensory characterization in terms of Appearance, firmness, smoothness, mouth‐ feel, flavor/taste, and overall acceptance was determined with the “Multiple Comparison Technique” by 10 panelists. Results were recorded by applying a hedonic scale (from 1 to 9) for various properties (Shah et al., 2010).

### 3.14 Statistical analysis

All tests were carried out as independent triplicates, and the outcomes were shown as mean and standard deviation. Data were assessed by analysis of variance (ANOVA) followed by Tukey's post test (95% confidence interval), applying the SPSS versio28 (Silva et al., 2022).

## 4 RESULT AND DISCUSSION

### 4.1 Characterization of microcapsules

The size distribution and zeta potential of microcapsules were assessed. The uniformity of microcapsules is indicated by the span index. In the present study, the span index was equal to 1.3 ± 0.002. The polydispersity index (PDI) in the present research was 0.263 ± 0.1. Other researchers have affirmed various span index and PDI for different microcapsules fabricated with different wall materials and different microcapsule preparation approaches (He et al., 2020).

Results showed that the zeta potential and mean mobility of microcapsules were −28.17 mV and −2.23 μm s^−1^ V^−1^ cm^−1^, respectively. Greater zeta potential related to the higher repulsive force between drops and a less tendency to adhere to each other and greater resistance of emulsion (Mao et al., 2009).

### 4.2 SEM

**FIGURE 1:**
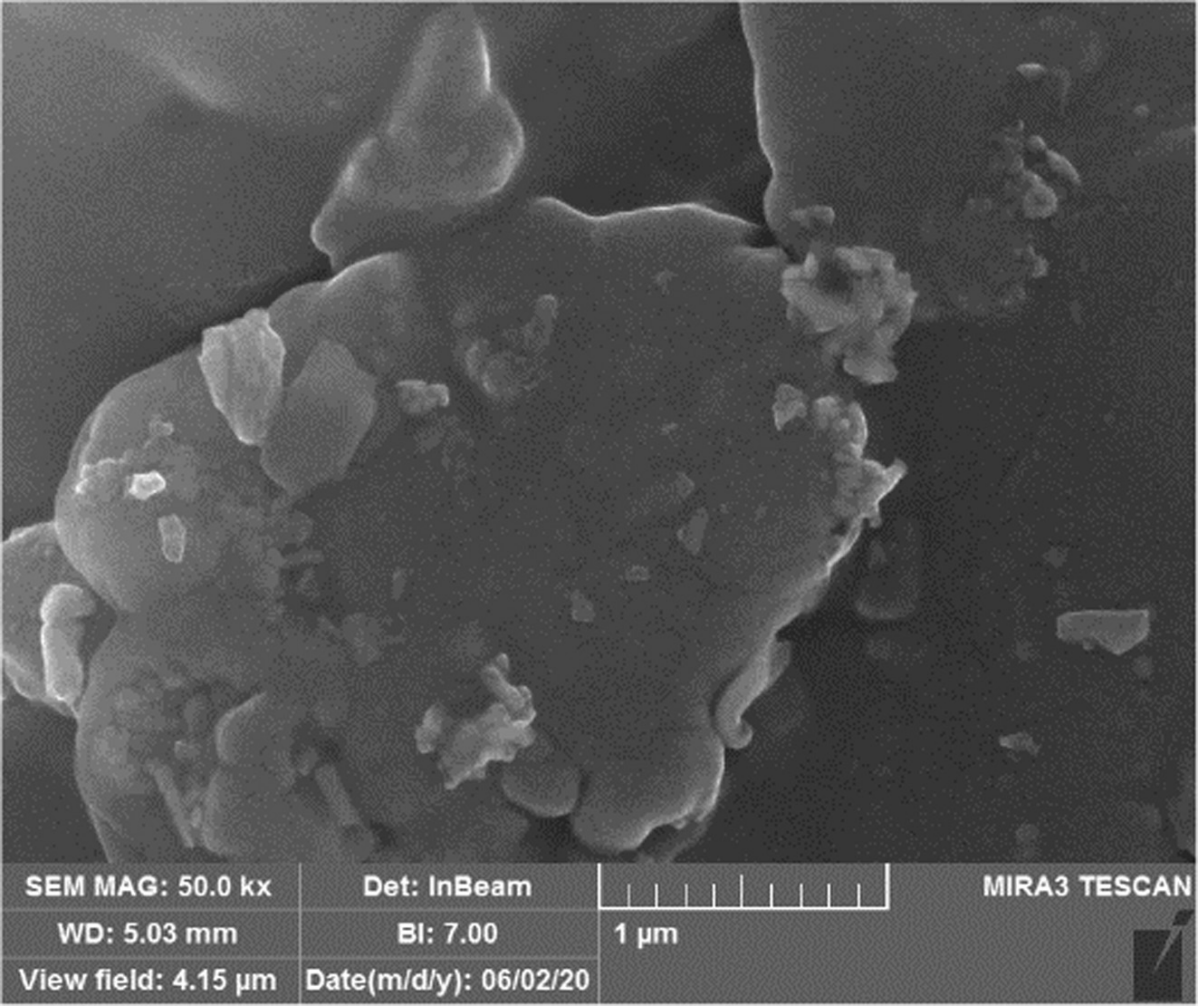
SEM image of microcapsules.

The formation of microcapsules was affirmed by Scanning Electron Microscopy (SEM) at a magnification 50.0kx. The observed microcapsules are shown in Figure 1.

According to the SEM image, the production of microcapsules was affirmed. These outcomes are close to the previous reports where various cells were encapsulated in various polyelectrolytes and polymers that indicated cellular morphologies and functionalities (Anwar et al., 2022).

### 4.3 Encapsulation efficiency of microcapsules in terms of probiotic, TPC, and TAC

After calculation of the encapsulation efficiency of TPC, TAC, and probiotic cells, there were equal to 90.91%, 89.62%, and 95.5%, respectively. Kanha et al. (2021) reported chitosan‐carboxymethylcellulose (CS‐CMC) microcapsules with microencapsulation efficiency magnitudes equal to 87.6%–94.7%, which were greater than those of microcapsules produced with gelatin‐acacia gum (GE‐AG) (84.9%–90.4%) (*p* ≤ .05; Kanha et al., 2021). Afzaal et al. (2022) approved that the encapsulation efficiency for *Bifidobacterium bifidum* encapsulated in sodium alginate (SA) or whey protein isolate (WPI) was 99% and 95%, respectively.

### 4.4 Characterization of chocolate samples

Several properties of chocolate samples (control and enriched ones) were analyzed including particle size distribution, thermogravimetric analysis of chocolate samples, total phenolic content, antioxidant activity of chocolate samples, Raman spectra, color, and rheological properties.

### 4.5 Thermogravimetric analysis of chocolate samples

**FIGURE 2:**
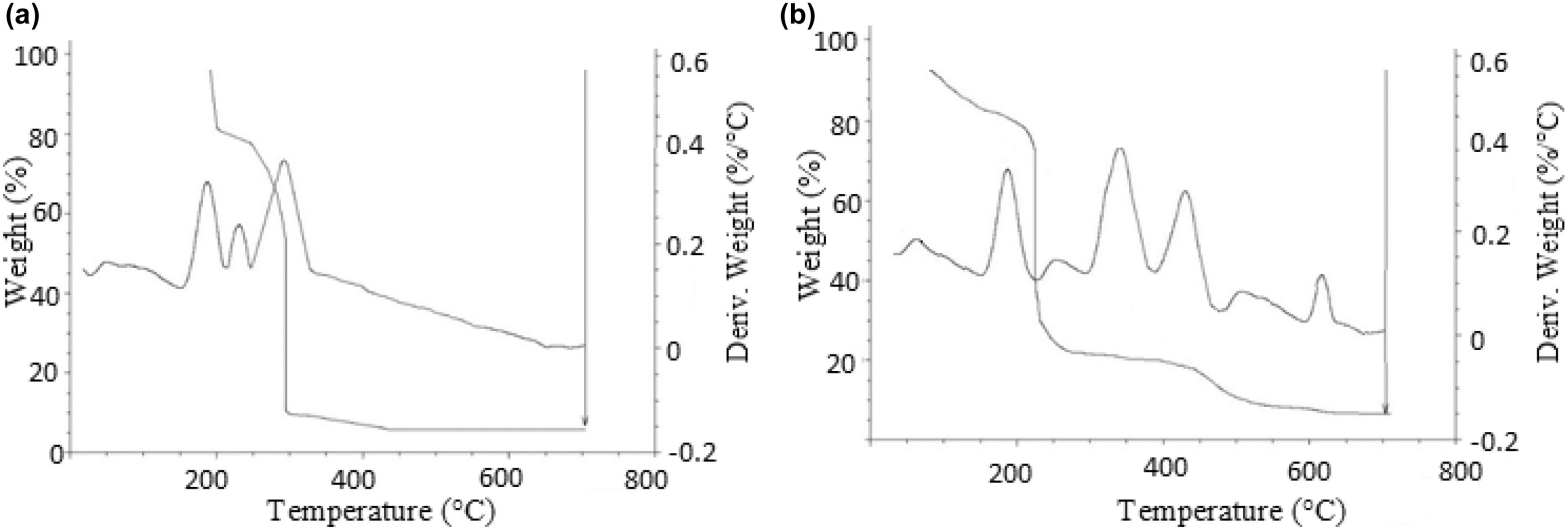
TG and DTG curves of white chocolates. (a) Control, (b) enriched white chocolate.

Thermogravimetric curves were assessed and the first derivative (DTG) for all samples was determined. In Figure 2a,b, TG and DTG curves of white chocolates (control and enriched sample) with heating rates 10°C min^−1^ are depicted. Materazzi et al. (2014) affirmed that heating rate of 10°C min^−1^ is the most desirable resolution for such tests (Materazzi et al., 2014).

The shapes of TG curves of the control sample showed the first transition appeared in the range of 50–230°C, the second one at 230–300°C, and the third one at 300–700°C. The mass loss happened with a high rate for control white chocolate rather than for enriched one (Figure 2a,b). According to the DTG curves, the prior peaks' maximum temperature range is related to the thermal degradation of sugar on all of the DTG curves (Figure 2a,b). The second transition might be attributed to cocoa butter oxidation. The DTG curves for the control sample showed a peak at a maximum temperature range from 291 to 294°C. The DTG curve of enriched white chocolate was composed of more transition peaks than the control sample (72.49, 187.40, 208, 340.52, 441.81, 513.35, and 609.85°C). A faster mass loss was seen between 180 and 220°C, during which the organic components, possibly phenolics, burn. Due to the complicated action of melted sugars, thermal degradation reactions could happen prior or close to the melting point, and in the reports, a range of magnitudes for sucrose melting differs between 185 and 190°C, yet it never reaches 225°C (Ducat et al., 2015). Ostrowska‐Ligęza et al. (2018) stated four different transitions for cocoa butter including the prior stage of 50–290°C, the second stage of 290–335°C, the third stage of 335–420°C, and the fourth stage of 420–700°C attributed to polymorphism of cocoa butter.

Four steps of degradation on TG and DTG curves of cocoa liquor at maximum temperatures of 253, 320, 447, and 491°C were depicted which attributed to the composition of the cocoa liquor which is a mixture of cocoa butter, cocoa powder, cocoa solid, antioxidant flavor, and mineral compounds. The temperature of degradation was related to the thermal decomposition of the constituents of cocoa liquor. The DTG curve depicted a peak at a maximum temperature of 518°C, which implies the sugar sample degradation.

### 4.6 Thermal properties, particle size distribution, and rheological characterization of chocolate samples

Based on the outcomes, the inclusion of microcapsules in white chocolate composition causes a change in the thermal, rheological as well as particle size of chocolate samples (Table 1).

**TABLE 1:** Thermal properties, particle size distribution, and rheological characterization of chocolate samples.

Sample	*T*_onset_ (°C)	*T*_peak_ (°C)	Δ*H* (J g^−1^)	d (0.1)	d (0.5)	d (0.9)	Casson viscosity (pas)	Casson yield stress (Pa)
Control	28 ± 0.5^b^	33.2 ± 0.2^b^	38.1 ± 0.1^a^	2.34 ± 0.03^a^	8.21 ± 0.02^a^	20.29 ± 0.01^a^	1.25 ± 0.5^a^	4.6 ± 0.01^a^
Enriched Sample	29.2 ± 0.4^a^	34.1 ± 0.3^a^	36.2 ± 0.4^b^	2.33 ± 0.06^a^	8.65 ± 0.03^b^	23.6 ± 0.01^b^	1.54 ± 0.03^b^	4.56 ± 0.02^ab^

*Note*: Different superscript lowercase letters depict significant differences between the samples (*p* < .05).

Abbreviations: *T*_onset_, onset temperature; *T*_peak_, peak temperature; Δ*H*, the energy required for the complete melting of the samples.

The magnitudes of onset (*T*_onset_), peak (*T*_peak_), and melting enthalpy (Δ*H*_melt_) magnitudes are depicted in Table 1. Accordingly, for the control sample, *T*_onset_, *T*_peak_, and Δ*H*_melt_ were 28 ± 0.5, 33.2 ± 0.2, and 38.1 ± 0.1, respectively. In the enriched sample, the magnitude of *T*_onset_ and *T*_peak_ reached 29.2 ± 0.4 and 34.1 ± 0.3a, respectively. These values were in accordance with values reported for chocolate fortified with microencapsulated fish oil reported (Hadnađev et al., 2023). In comparison with the control chocolate, the inclusion of microcapsules led to higher onset and peak temperatures of the enriched chocolate. According to the results presented in Table 1, the control sample had a greater magnitude of melting enthalpy (Δ*H*_melt_) rather than the samples including microcapsules. Similar behavior was reported by Hadnađev et al. (2023) who ascended the greater melting enthalpy in the control sample to the firmer structures in the control chocolate rather than the enriched sample with microcapsules (Hadnađev et al., 2023).

Rheological parameters are presented in Table 1. Rheological measurements, approve that the values of the Casson plastic viscosity of the enriched sample were remarkably higher than the control sample. These outcomes are in accordance with the outcomes of Hadnađev et al. (2023) who affirmed the addition of fish oil microcapsules resulted in increasing the Casson plastic viscosity and ascending to that addition of microcapsules causes greater solid particle amount and diminished the magnitude of fat phase (Hadnađev et al., 2023). However, no differences were observed in the samples containing microcapsules compared to the control one in terms of Casson yield stress (Pa; Table 1).

The particle size distribution of samples was determined to assess chocolates with and without microcapsules. Table 1 indicates the impact of the addition of microcapsule on the particle size parameters D_10_, D_50_, and D_90_. Based on the outcomes, it can be obvious that the microcapsule inclusion remarkably influenced the particle size distribution parameters except D_10_. The major remarkable enhancement in the size values with the inclusion of microcapsules was seen for D_50_ and D_90_. Although, the D_90_ was lower than 25 μm for enriched the sample. This was a desirable outcome on the basis that the maximum particle size for desirable chocolate should be lower than 35 μm (Tolve et al., 2018).

### 4.7 Characterization of antioxidant activity, TPC, TAC, hardness, and color indices of white chocolate

Antioxidant activity, TPC, TAC, hardness, and color indices of white chocolate were measured and the results shown in Table 2.

**TABLE 2:** Antioxidant activity, TPC, TAC, hardness, and color indices of samples.

Sample	Antioxidant activity (%)	TPC (mg/100 g)	TAC (μg/100 g)	Hardness (g)	L*	a*	b*
Control	0^b^	0.11 ± 0.01^b^	0^b^	4145.32 ± 52^b^	55.5 ± 0.03^a^	5 ± 0.01^b^	3.42 ± 0.02^a^
Enriched Sample	38 ± 2%^a^	2.1 ± 0.2^a^	2.03 ± 0.1^a^	4361.22 ± 82^a^	32 ± 0.02^b^	20 ± 0.002^a^	3.13 ± 0.02^b^

*Note*: Different superscript lowercase letters depict significant differences between the samples (*p* < .05).

L* represents darkness to lightness, a* represents greenness to redness and b* represents blueness to yellowness.

Color parameters in terms of L*, a*, and b* were measured by MINOLTA Chroma Meter CR‐400 and the outcomes are depicted in Table 2. Accordingly, the inclusion of microcapsules affects all the color parameters (*p* < .05). The control sample has a higher L* which implies more lightness of the control sample than the enriched one. The magnitude of a*(redness) is also enhanced as the microcapsules were included in the chocolate sample (Table 2). This observation could be due to the existence of several anthocyanin compounds in the rose hip fruit shell (Bozhuyuk et al., 2021); Kayahan et al. (2022) reported the amount of carotenoids (mg/100 g DW) of rose hip from *R. canina* was 1356.73 ± 27.2. Total carotenoid amount of various rose hip species was determined to be between 1204.5 (*R. canina*) and 1235.7 (*R. sempervirens*) by Fascella et al. (2019).

Measurement of textural property in terms of the harness was carried out by A.XT plus texture analyzer (Stable Micro Systems) and the outcomes are indicated in Table 2. Considering the outcomes, the inclusion of the microcapsules resulted in increasing the hardness of samples (*p* < .05). This result is fitted with the report of Lončarević et al. (2018) who attributed the higher hardness of enriched chocolate with encapsulated blackberry juice to a more compact structure owing to the low magnitude of free fat phase in the enriched sample. Several parameters, including formulation, production circumstances, tempering, and fat crystal polymorphism, could influence the stiffness of chocolate. Stiffness is one of the characteristics that implies the degree of desirable tempering and the establishment of the fat crystal network. The stiffness of chocolate is affected by the crystallization of cocoa butter, crystal size, morphology, and polymorphic trend (Genc Polat et al., 2020).

The anthocyanin content of the control sample was equal to zero but in the enriched sample the content of total anthocyanin was determined as 2.03 μg/100 g (Table 2).

In the present research, the amount of total anthocyanin in the rosa hip fruit shell was equal to 4.8 mg kg^−1^. According to the Bozhuyuk et al. (2021), the total anthocyanin content of fruits of unsprayed *Rosa canina* was between 3.62–7.81 mg kg^−1^.

### 4.8 Raman spectroscopy of chocolate samples

**FIGURE 3:**
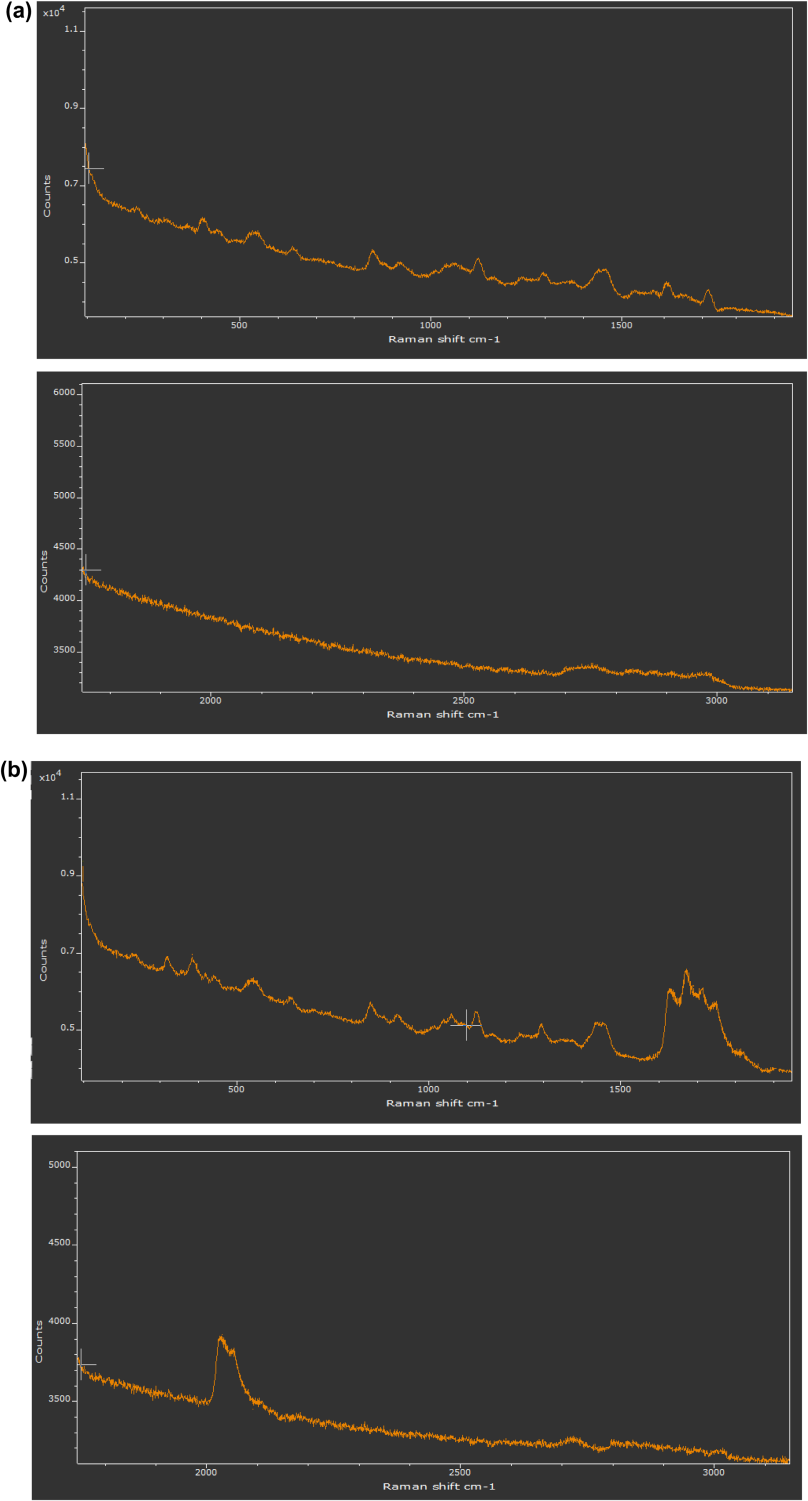
Raman spectra of control chocolate sample (a) and chocolate included microcapsules (b).

The Raman spectra of control and enriched white chocolate were shown in Figure 3a,b.

In the case of control, white chocolate, the spectral region (Raman shift cm^−1^) at 600–900 (401, 408, 534, 541, and 548 cm^−1^) attributed to sucrose (Raman spectroscopy at 1000 nm for chocolate measurements). The peaks observed at (1420–1480 cm^−1^) attributed to the cocoa butter, sugar, saturated fatty acids in cocoa butter and sucrose (Raman spectroscopy at 1000 nm for chocolate measurements, n.d.). Peaks appeared at 1650–1670 and 1700‐1780 cm^−1^ also could be ascended to the presence of cocoa butter (Raman spectroscopy at 1000 nm for chocolate measurements, n.d.).

In the first region of spectra of enriched chocolate, the peak observed at 2008 cm^−1^ is ascending to the stretching vibration of the hydroxyl (O‐H) in the A, B, and C rings of anthocyanins. The peak at 1638 cm^−1^ is related to the stretching of aromatic C=C, implying the existing presence of anthocyanins in the microcapsules. The peak observed at 1378 cm^−1^ might be due to the bending of ‐CH_3_, whereas the peak at 1338 cm^−1^ implies the existing flavanol quercetin, and ascending to the in‐plane O–H bends of the C_3_‐OH group in C ring (Teslova et al., 2007). Flavonoids are also depicted by the stretch of C‐C in the aromatic ring, appeared in the peak at 997 cm^−1^. The peak at 972 cm^−1^ is ascending to the stretching of C=C in conjugated C‐C=C‐C categories, specified to various phenolic components. The peak observed at 862 cm^−1^ and in the range 800–700 cm^−1^ are owing to the aromatic C‐H out‐of‐plane bending vibrations, particularly in ring A. The peak at 628 cm^−1^ implies C‐C deformation. The peak at 395 cm^−1^ is related to the bending of C‐OH, whereas the peak at 344 cm^−1^ depicted C‐O stretching in all aromatic rings (Dranca & Oroian, 2019).

The peaks that appeared at 1515–1535 and 1185 cm^−1^ could be ascending to carotenoids (Saletnik et al., 2022). There are flavonoids component found in *Rosa canina* (shell) including Hyperoside, Rutin, Quercetin, Catechin, Astragalin. Some carotenoid components such as lutein and zeaxanthin are also ingredients of rose hip (Winther et al., 2016).

### 4.9 Survival ability of probiotic bacteria in white chocolates during storage

**FIGURE 4:**
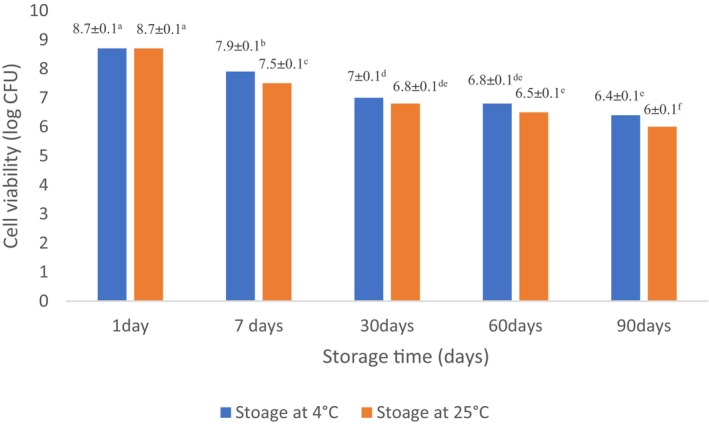
Survival of probiotic bacteria in chocolates on storage at 4 and 25°C for 90 days. Experiments were performed in triplicate and repeated three times. Values display mean ± SD. Different lowercase letters indicate significant differences at *p* < .05.

The white chocolate samples were assessed for the survival ability of probiotic bacteria in various storage times (until 90 days) and different storage temperatures (at 4 and 25°C; Figure 4). Observations imply that both temperature and duration of storage had a significant impact on the survival rate of probiotic bacteria. The primary magnitude of *L. acidophilus* in chocolates was 8.7 log CFU g^−1^, but the survival rate of bacteria was gently diminished in both storage temperatures as the storage period prolonged at 90 days storage at 4 and 25°C, the log of viable cells reached to 6.4 and 6, respectively (Figure 4). This observation was in accordance to Kobus‐Cisowska et al. (2019) who investigated the viability of *B. coagulans* in the chocolate carrier and reported during refrigeration circumstances, the reduction in viable bacteria was lower.

Islam et al. (2022) reported cell viability of *L. acidophilus* LDMB‐01 gradually declined in the samples which were refrigerated and the count strongly diminished in samples stored at 25°C as the storage time lengthened.

The major parameters related to the detriment of survival rate of probiotic bacteria include oxygen exposure, a reduction in pH, and the primary metabolism of lactic acid (Islam et al., 2022). After 90 days of storage at both temperatures (4, 25°C), the viable probiotic bacteria maintained at the desirable amount for probiotic‐enriched foods stated by Chaikham (2015) and Lalicic‐Petronijevic et al. (2015), who proposed that prior to consuming, at least 10^6^ CFU mL^−1^ or CFU g^−1^ of live probiotic bacteria should be existing.

In contrast, Begum et al. (2019) reported that the survival rate of *Lactobacillus acidophilus* NIAI L‐54 in watermelon juices with whey base stored at 4°C for 21 days, was 4 log CFU mL^−1^, and reported this observation was due to the type of selected food matrix.

### 4.10 Stability of anthocyanins and phenolic components in white chocolates during storage

**FIGURE 5:**
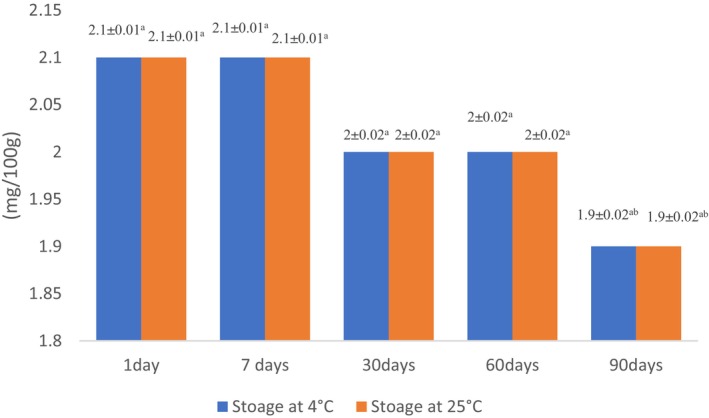
Stability of phenolic components in white chocolates during storage. Experiments were performed in triplicate and repeated three times. Values display mean ± SD. Different lowercase letters indicate significant differences at *p* < .05.

The total phenolic content change during various storage times at 4 and 25°C was determined and the results are depicted in Figure 5. Accordingly, there was a negligible reduction in TPC during storage but not statistically significant (*p* > .05). This observation is in accordance with the findings of Kobus‐Cisowska et al. (2019) that reported there was no significant decrease in TPC of dark chocolate samples up to 3 months of storage at ambient temperature.

**FIGURE 6:**
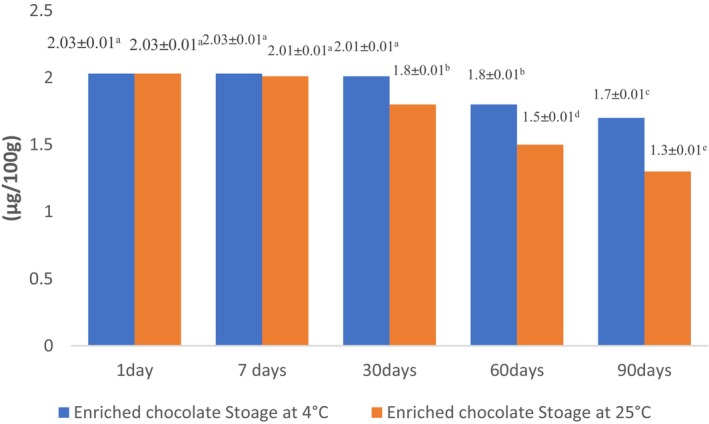
Stability of anthocyanins in white chocolates during storage. Experiments were performed in triplicate and repeated three times. Values display mean ± SD. Different lowercase letters indicate significant differences at *p* < .05.

In the case of anthocyanin stability at different storage temperatures (4 and 25°C), observation implies that either storage time and storage temperature have a remarkable impact on the anthocyanin amount of enriched chocolate (*p* < .05). This finding is consistent with Enache et al. (2022) who reported the magnitude of encapsulated anthocyanin from cornelian cherry (*Cornus mas* L.) fruits and lactic acid bacteria reduced during storage (from 32.14 ± 0.97 at 0 days of storage to 10.64 ± 0.66 at 90 days of storage). Accordingly, the magnitude of anthocyanin reduction was 67% (Enache et al., 2022). The stability of anthocyanin was significantly higher at lower storage temperature (4°C; Figure 6). Azarpazhooh et al. (2019) investigated the destruction kinetic of anthocyanin in a microencapsulated pomegranate peel extract during various storage circumstances (4°C and 25°C and RH = 52 and 75%), and concluded a greater stability of anthocyanin at a lower temperature.

Gültekin‐Özgüven et al. (2016) pointed out % loss of anthocyanin in samples encapsulated in chitosan‐coated liposomes was significantly lower and concluded that encapsulation of extract in chitosan‐coated liposomes guard anthocyanin.

### 4.11 The viability rate of *Lactobacillus acidophilus* under simulated gastrointestinal circumstances

**FIGURE 7:**
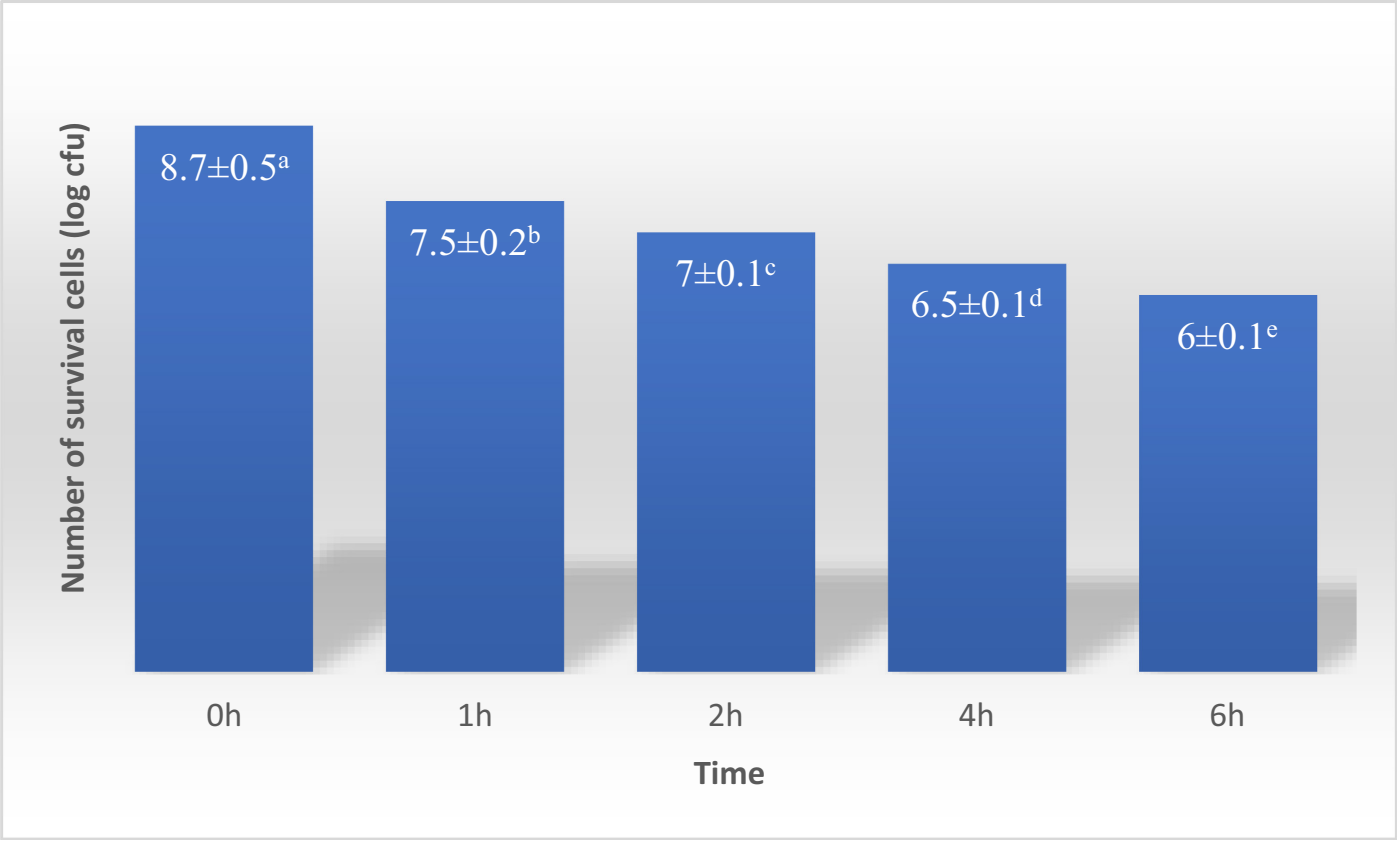
Release of probiotic bacteria as a function of time during gastric (SGF) and intestinal (SIF) in vitro digestion. Experiments were performed in triplicate and repeated three times. Values display mean ± SD. Different lowercase letters indicate significant differences at *p* < .05.

Within simulated gastrointestinal circumstances, the survival rate of *L. acidophilus*, in two chocolate matrixes, was assessed (Figure 7). Initially, the number of survival cells in simulated stomach fluid was 8.7 log CFU/10 g. As the time of incubation increased, the number of survival cells gradually decreased (*p* ≤ .05) and at the end of exposure to gastric simulation time, the survival cell number reached 7 log CFU mL^−1^. Islam et al. (2022) reported the survival of cells which were not encapsulated was less under simulated gastric circumstances, and they were completely destroyed after 2 h (Islam et al., 2022). In comparison, the number of survivals in the present study was greater which could be attributed to the encapsulation of probiotic bacteria that protect cells from the in vitro gastrointestinal parameters such as acidity, enzymes, incubation time, and oxygen level (Naissinger da Silva et al., 2021). Chocolate matrix also shows a protective role against gastric conditions as Islam et al. (2022) reported the cell survival of *L. acidophilus* has adequate survivability than free cells (Islam et al., 2022). After gastric digestion, the *L. acidophilus* cells were incubated in the simulated small intestinal fluid for the next 4 h and their viability at the end of 2 h reaches 6 log CFU. The desirable survivability could be attributed to the encapsulation of probiotic bacteria as well as the chocolate matrix that has a protective impact against unfavorable circumstances (Islam et al., 2022).

This outcome is consistent with the outcomes of Bakhtiyari et al. (2022), who stated the survival rate of *L. plantarum*, which was not encapsulated, was missing rapidly in SGF as the number of cells diminished. A greater content of WPI (2.76%) depicted the better guard impact on the cells (*p* ≤ .05) in SGF and explained that a greater WPI amount caused the formation of an adhesive dense gel network that diminished the permeation of H^+^ into the beads (Bakhtiyari et al., 2022). Another reason for more stability and gentle reduction in cells in encapsulated ones is the extremely acidic endurance and great buffering characteristics of WPI (Bakhtiyari et al., 2022).

The release of polyphenolic components also is a contributing parameter in the survivability of probiotic cells. Molan et al. (2009) stated polyphenols found in green tea (antiradical constituent) balanced oxidative stress shortcoming by cellular metabolic activities, so phenolic extract might provide a more desirable condition for the cell multiplication under acidic circumstances (Molan et al., 2009). Belščak‐Cvitanović et al. (2016) stated that the multiplex hydroxyl groups of polyphenols imply strong interactions with CS amino groups via hydrogen and covalent bonds which limit the release of polyphenols from the beads on test duration (Belščak‐Cvitanović et al., 2016).

One of the major reasons for encapsulation is the diffusion of encapsulated probiotics in the intestine (Shi et al., 2013). The log of the viable cell at the beginning stage was 8.7 and after 1 h reaches 7.5. The reduction of viable cells gradually continued and at the finish stage of the intestinal simulated condition, the log of viable cells was equal to 6. Desirable cell viability in the present research could be attributed to the encapsulation protection impact against acid and enzyme hydrolysis in simulated gastrointestinal conditions as well as the presence of the phenolic components having a direct impact on cell viability.

Silva et al. (2022) reported the magnitude of probiotic bacteria release from coacervates at SGF was equal to 45% and attributed to the adherence of probiotics to the surface of coacervates; the hydrophilicity of probiotics as well as low pH and pepsin may comfort the release of probiotics, as explained by the enhancement of viable probiotics in SGF after 120 min of the in vitro experiment. According to their study, co‐encapsulation of probiotic bacteria and guarana peel or seed extract diminishes the release of probiotics at SGF and they interpreted this observation as probably increasing and reducing the untimely release of probiotics in SGF. A rapid release of probiotics in SGF is not favorable because it could result in probiotic death under severe circumstances. So, the existence of phenolic components could be suitable for retaining the viability of untimely released probiotics in the middle, depicting a possible benefit of co‐encapsulating probiotics with phenolic components from plant extracts (Silva et al., 2022).

### 4.12 Release of total phenolic content and total anthocyanin content in vitro digestion

**FIGURE 8:**
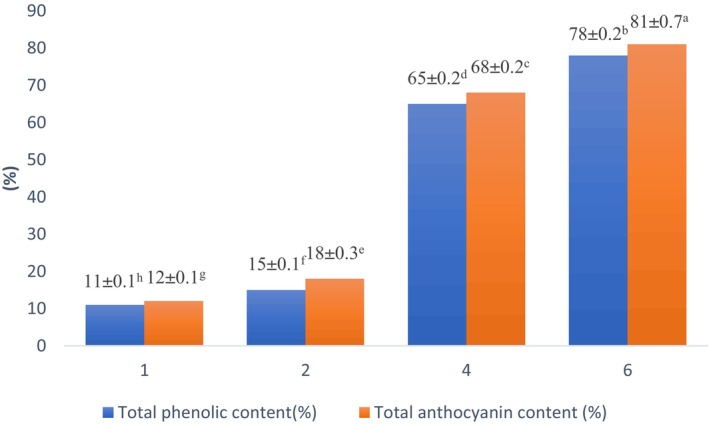
Release of total phenolic content and total anthocyanin content (%) as an action of time during gastric (SGF) and intestinal (SIF) in vitro digestion. Experiments were performed in triplicate and repeated three times. Values display mean ± SD. Different lowercase letters indicate significant differences at *p* < .05.

The amount of released TPC and TAC was determined during exposure to SGF (pH 1.5) and SIF (pH 6.8). The magnitude of TPC and TAC release at a time of 1 h subjecting to the simulated gastric condition were 11% and 15%, respectively. After 2 h of exposure to gastric condition, the release rate of TPC and TAC reaches 15% and 18%, respectively. Generally, all over the 300 min, the release of anthocyanins and phenolic components enhanced but the rate of release for both component groups in simulated gastric condition was significantly lower than in simulated intestinal condition (Figure 8). Observation showed a similar trend of phenolic components release in SGF and SIF condition. At the start of the SGF condition, the release of phenolic components was 11% and reaches 15% after 2 h. The intestinal fluid exposure caused the release of about 78% of phenolic compounds and 81% of anthocyanin content (Figure 8).

The anthocyanin release trend affirmed that the structure of microcapsules (porosity) caused diffusion of anthocyanin components to out of the microcapsules. The magnitude of released phenolic compounds and anthocyanins to the gastric phase were 15% and 18%, respectively. This is fitted with the report of Seke et al. (2022) who affirmed the magnitude of anthocyanin release to the gastric phase in alginate beads included phenolic extract of natal plum (*Carissa macrocarpa*) was 18% (De Cássia Sousa Mendes et al., 2021). During the intestinal phase, the anthocyanin content which was released from the microcapsules was enhanced (Figure 8). Under incubation in SIF (2 h), the magnitude of anthocyanins released was 68% and at the finished step of intestinal phase exposure, the magnitude of anthocyanin released reaches to 81% (Figure 8). These values are greater than the report of Seke et al. (2022) that the amount of anthocyanin released in intestinal condition was 24%–29%. In this respect, it could be ascending to the material structure of microcapsules as Łupina et al. (2021) stated that the release of Astaxanthin from the matrix with more swelling capacity (Gum Arabic/Gelatin) was more rapid and attributed to the different swelling rates of films (Łupina et al., 2021).

Generally, there is a reverse relationship between the drug diffusion magnitude and the swelling capacity of the matrix, owing to the entrapment of the drug in a swollen network, therefore, enhancing its diffusion rate (Łupina et al., 2021).

The magnitude of recovery and the bioaccessibility of anthocyanin and phenolic components were calculated according to Seke et al. (2022) and the outcomes are indicated in Table 3. Considering the outcomes, the bioaccessibility of anthocyanin and phenolic components was 81% and 78%, respectively. These magnitudes are close to the magnitudes reported by Hossain et al. (2022) who affirmed the bioaccessibility of polyphenols (83.22%–92.33%) in chocolates fortified with encapsulated probiotics (Faccinetto‐Beltrán et al., 2021).

**TABLE 3:** The influence of gastrointestinal digestion on the anthocyanin and total phenolic recovery and bioaccessibility of enriched chocolate.

TAC microgram 100 g^−1^ undigested	2.03	TPC mg 100 g^−1^ undigested	2.1
TAC microgram 100 g^−1^ gastric digestion	0.36	TPC mg 100 g^−1^ gastric digestion	0.315
Recovery %	18%	Recovery %	15%
TAC microgram 100 g^−1^ intestinal digestion	1.64	TPC mg 100 g^−1^ intestinal digestion	1.638
Bio accessibility %	81%	Bio accessibility %	78%

Hossain et al. (2022) reported during the in vitro gastrointestinal digestion, polyphenol bioaccessibility was associated with the existence of probiotic bacteria in the chocolate samples and concluded that their interaction caused an increase in bioaccessibility under in vitro gastrointestinal digestion.

Dala‐Paula et al. (2021) pointed out the better polyphenol bioconversion abilities in samples with more number of added probiotics (Hossain et al., 2022). A probable interpretation is that the high magnitude of polyphenols was capable of diminishing the oxidative stress that happens under gastrointestinal circumstances, which resulted in probiotic death (Dala‐Paula et al., 2021). The improvement of bioaccessibility and bioavailability of bioactive components with the inclusion of probiotics to chocolates is also affirmed by animal models or clinical studies (Yang et al., 2020).

### 4.13 Assessment of the microstructure of chocolate samples

**FIGURE 9:**
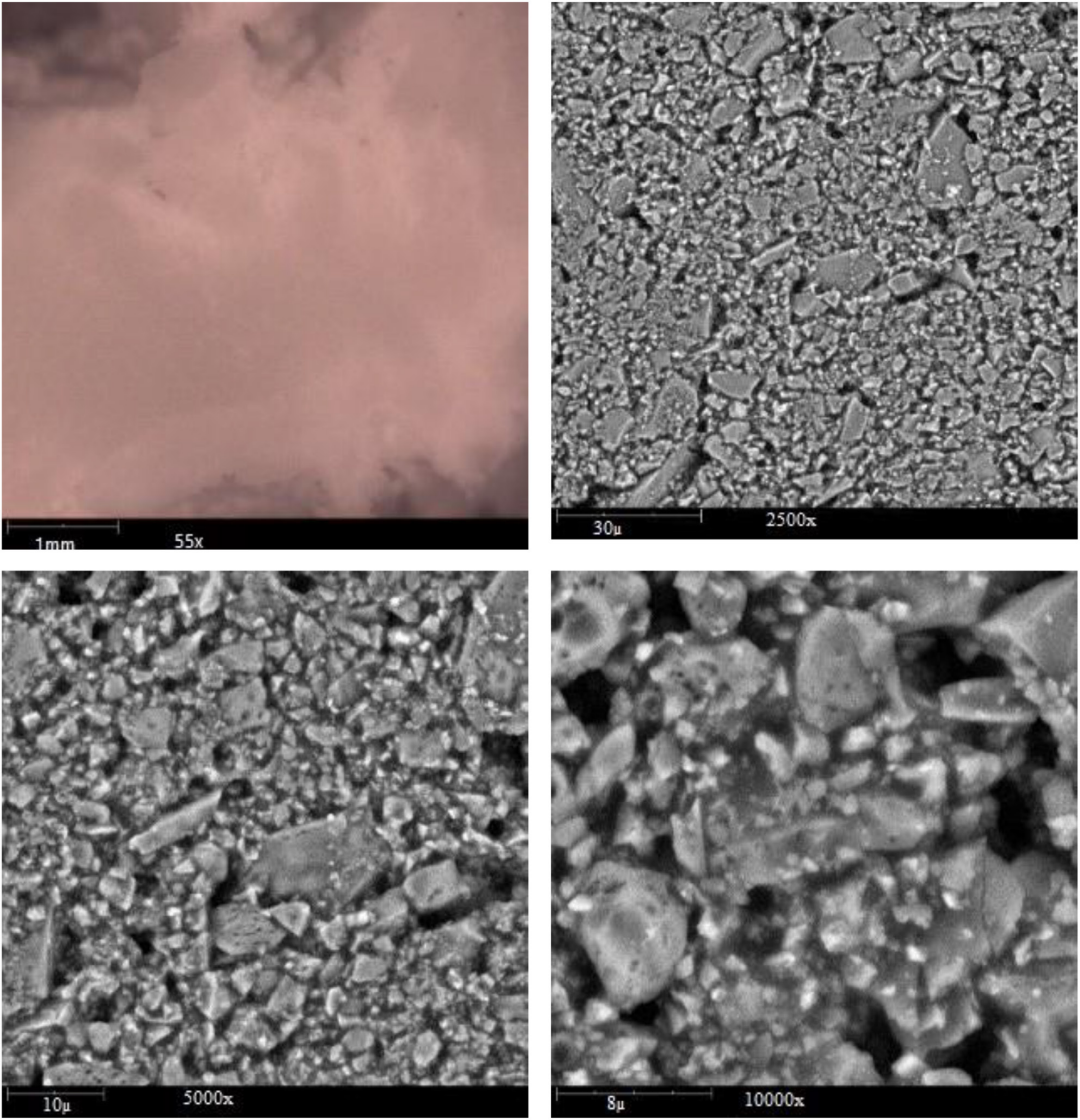
SEM observation of control chocolate.

**FIGURE 10:**
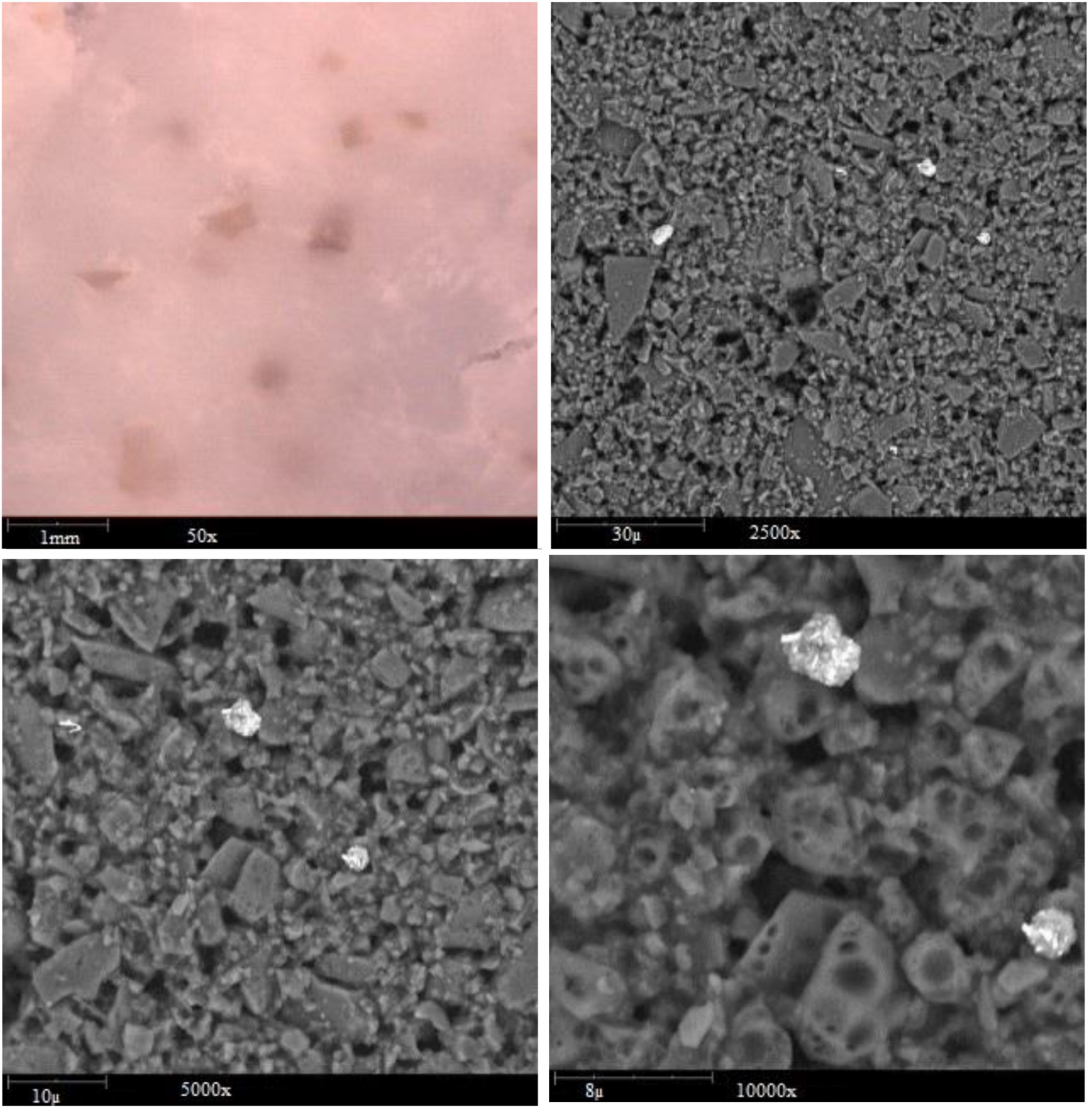
SEM analysis of enriched chocolate.

Assessment of the microstructure of chocolate samples was accomplished by applying SEM (phenom, proX model), and the images were presented in Figures 9 and 10. Accordingly, both chocolate samples have a compact structure. The presence of microcapsules was observed in the enriched chocolate sample (Figure 10).

### 4.14 Sensory evaluation of chocolate samples

**FIGURE 11:**
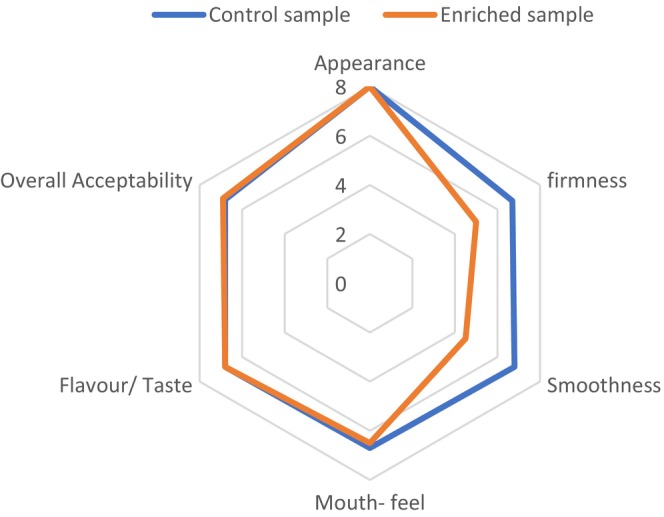
Sensory properties of white chocolate samples.

Organoleptic evaluation was performed with an untrained, consumer panel using hedonic scaling from 1 to 9. The attributes determined are composed of appearance, smoothness, firmness, mouthfeel, flavor/taste, and overall acceptance (Shah et al., 2010). The outcomes are depicted in Figure 11. Accordingly, there was a remarkable difference between control and enriched chocolate samples in terms of appearance, firmness, and smoothness but the overall acceptance and flavor/ taste of chocolate samples have no remarkable difference (*p* < .05).

The existence of carotenoids and various phenolic components in rose hip fruit is reported by various studies (Kayahan et al., 2022), these components affect the appearance, mouth‐feel, and flavor and taste of enriched chocolate. According to the sensory evaluation of chocolate samples, the firmness of enriched chocolate was higher than the control sample. This is fitted with the result of the measurement hardness of chocolate samples and the magnitude of the hardness of enriched chocolate was more than the control one (Table 2). Finally, the overall acceptance of samples was no remarkable difference and the magnitude of acceptance of both chocolate sample was similar (Figure 11).

## 5 CONCLUSION

This study is focused on producing a novel type of probiotic chocolate including co‐encapsulated *Lactobacillus acidophilus* bacteria and rose hip shell fruit extract. The addition of microcapsules to chocolate affects its quality as measured by rheological, textural, and thermal properties, particle size distribution, color indices, total phenolic content, total anthocyanin content, antioxidant activity, and Raman Spectroscopy. The outcomes of this study imply that white chocolate is a desirable matrix for *Lactobacillus acidophilus* probiotic bacteria, and the sensory properties of probiotic chocolate are acceptable. The great viability of bacteria incorporated in chocolate and the stability of phenolic components and anthocyanins under simulated digestion circumstances in the gastrointestinal tract and during storage were also observed.

## AUTHOR CONTRIBUTION

**Zohreh Didar:** Conceptualization (equal); data curation (equal); formal analysis (equal); funding acquisition (equal); investigation (equal); methodology (equal); project administration (equal); resources (equal); software (equal); supervision (equal); validation (equal); visualization (equal); writing – original draft (equal); writing – review and editing (equal).

## FUNDING INFORMATION

The author acknowledges the Iran High‐Tech Laboratory Network for partial financial support of this research.

## CONSENT TO PARTICIPATE

The author read and approved the final manuscript.

## CONSENT FOR PUBLICATION

The author has read and agreed to the published version of the manuscript.

## Data Availability

Data are available upon request.
